# Hind limb unloading of mice modulates gene expression at the protein and mRNA level in mesenchymal bone cells

**DOI:** 10.1186/1471-2474-11-147

**Published:** 2010-07-05

**Authors:** Davide Visigalli, Antonella Strangio, Daniela Palmieri, Paola Manduca

**Affiliations:** 1 Genetics, DIBIO, University of Genoa, (Corso Europa 26), Genoa, (I-16132), Italy; 2 DIMES, University of Genoa, (Via De Toni), Genoa, (I-16132), Italy

## Abstract

**Background:**

We investigated the extent, modalities and reversibility of changes at cellular level in the expression of genes and proteins occurring upon Hind limb unloading (HU) in the tibiae of young C57BL/6J male mice. We focused on the effects of HU in chondrogenic, osteogenic, and marrow mesenchymal cells.

**Methods:**

We analyzed for expression of genes and proteins at two time points after HU (7 and 14 days), and at 14 days after recovery from HU. Levels of mRNAs were tested by in situ hybridization. Protein levels were tested by immunohistochemistry. We studied genes involved in osteogenesis (alkaline phosphatase (AP), osteocalcin (OC), bonesialoprotein (BSP), membrane type1 matrix metalloproteinase (MT1-MMP)), in extracellular matrix (ECM) formation (procollagenases (BMP1), procollagenase enhancer proteins (PCOLCE)) and remodeling (metalloproteinase-9 (MMP9), RECK), and in bone homeostasis (Stro-1, CXCL12, CXCR4, CD146).

**Results:**

We report the following patterns and timing of changes in gene expression induced by HU: 1) transient or stable down modulations of differentiation-associated genes (AP, OC), genes of matrix formation, maturation and remodelling, (BMP1, PCOLCEs MMP9) in osteogenic, chondrogenic and bone marrow cells; 2) up modulation of MT1-MMP in these same cells, and uncoupling of its expression from that of AP; 3) transient down modulation of the osteoblast specific expression of BSP; 4) for genes involved in bone homeostasis, up modulation in bone marrow cells at distal epiphysis for CXCR4, down modulation of CXCL12, and transient increases in osteoblasts and marrow cells for Stro1. 14 days after limb reloading expression returned to control levels for most genes and proteins in most cell types, except AP in all cells, and CXCL12, only in bone marrow.

**Conclusions:**

HU induces the coordinated modulation of gene expression in different mesenchymal cell types and microenvironments of tibia. HU also induces specific patterns of expression for homeostasis related genes and modulation of mRNAs and proteins for ECM deposition, maturation and remodeling which may be key factors for bone maintenance.

## Background

Bone loss may be induced by immobilization of humans for long times in bed, and by hind limb unloading of rodents. HU allows ex vivo investigations into the processes and effects of weightlessness in long bones [1,2]. Effects of HU have been detected earliest on the viability of osteoblasts [3], followed by apoptosis of the osteocytes, activation of osteoclastogenesis and loss in bone mass, attributed to altered mechano-transduction by osteoblasts [4]. HU-induced changes in the expression of differentiation-associated genes have been reported in studies on whole bone tissue [5]. In ex-vivo experiments in rats, HU determined the decrease in number of colony forming cells from the bone marrow osteogenic compartment, and in AP expressing colonies [6].

The expression of differentiation-associated genes in osteoblasts and preosteoblasts is altered in simulated microgravity conditions, reproducing in vitro the condition of reduced mechanical stress occurring in space flight and in HU [7-9]. In microgravity, adipocyte differentiation is promoted from mesenchymal precursor cells [10] and collagen I synthesis is impaired in osteogenic and fibroblastic cells [7-9,11]. Osteopontin and parathyroid hormone receptor have been associated with the modulation of bone loss in HU conditions [12,13].

Studies are needed to illustrate the changes in gene expression at the level of individual mesenchymal cell types in the bone of HU animals, and to find more information on the expression of proteins. In addition, no data are available on the effects of HU on genes governing matrix deposition and remodeling, or on genes involved in controlling the homeostasis of the different cell types in bone. Little is known about the timing of the modulation in different cell types for each of the responsive genes by HU. Reversibility of these changes once weight is re-applied to the limbs is poorly understood.

In this paper we report investigations to start filling these gaps and suggest that valuable information can be obtained by studying the regulation by HU of proteinases and chemokine genes and proteins at cellular levels, in association with classic osteogenic markers.

We determined by in situ hybridization and/or immunohistochemistry the expression of individual genes and proteins in the tibiae of 11 week old male mice C57BL/6J, after HU for 7 and 14 days. We focus our report on gene expression in chondrocytes at the growth plate, osteocytes, endosteum and trabecular lining osteoblasts and in bone marrow cells.

Changes in the growth plate, and then in bone morphology, occurred after 3, 7 and 14 days and were documented. Bone loss was quantified by microcomputed tomography analysis (μCT) at 14 days. We also studied the expression of genes in tibiae of animals kept in HU for 14 days and then set on the ground for the next 14 days (re-loading). We compared same age control, and HU mice for the expression of key genes associated to the differentiation of osteoblasts (AP, OC, BSP), of genes involved in the deposition of ECM (BMP-1 and PCOLCE1 and 2) and in its remodeling (MT1-MMP, MMP-9, RECK), and of genes implied in bone homeostasis (Stro1, CD146, CXCR4 and CXCL12).

A decrease in AP and OC expression was previously described by examining mRNA from whole bones of HU rodents [14]. BMP1, mammalian tolloid (mTLD) and the genes for PCOLCE1 and 2, are expressed in modulated fashion during in vitro osteogenesis in rat osteoblasts (Manduca et al unpublished); the level of expression of their proteins controls the rate and amount of deposition of collagen fibers and the activation of pro-lysyl oxidase, also involved in the deposition of collagen fibrils [15,16]. PCOLCEs and BSP, known to be highly expressed by osteoblasts and osteoclasts [17], are proteins with a role in the mineralization of the ECM and PCOLCE1 is an important determinant of bone mechanical properties and of the geometry and morphology of collagen fibrils in mammals [18,19]. PCOLCEs gene expression decreases in osteoblasts cultured in microgravity [7].

Enzymes remodeling the ECM emerged as important for the osteogenic progression [20-22]. The major MMPs family members expressed by differentiating osteoblasts, MT1-MMP, MMP-2 and -9 are developmentally regulated during osteogenesis in vitro [21]. In rat osteoblasts differentiating in vitro, MT1-MMP expression affects the expression of pro-MMP-2 and of AP, and the activation of pro-MMP-2 pleiotropically [22]. MMP-9 modulation is not apparently associated with that of the other MMPs. RECK is an endogenous membrane inhibitor of MMPs [23], also modulated in its expression during osteogenesis in vitro [24].

CXCR4 [25] is the receptor for Stromal cell-derived factor-1 (SDF-1 or CXCL12), which is constitutively secreted by osteoblasts and bone marrow stromal cells. Dynamic levels of CXCL12 and CXCR4 expression play a key role in the homing of hematopoietic cells to the bone marrow, in the mobilization of circulating osteoblast precursors and in the recruitment of bone-resorbing osteoclasts, of osteoblasts, neutrophils, and other myeloid cells [26]. Activation of the CXCR4/CXCL12 pathway induces proliferation of hematopoietic and mesenchymal progenitors [27]. CXCL12 also stimulates mononucleate cell fusion and TRAP activity and is a key factor in the normal homeostatic regulation of bone development and remodeling [28]. CD146 is an endothelial, smooth muscle cell and pericyte marker [29] and Stro1 a stage- and/or lineage-specific stromal antigen [30].

Our results show that HU modulates the expression of the osteogenic differentiation markers AP, MT1-MMP, BSP and OC consistently in all the cells expressing these genes, even if of different differentiation lineages. Uncoupling of the associated expression for AP and MT1-MMP occurs in cells of the osteogenic lineage.

HU modulates proteolysis related genes suggesting that pericellular proteolysis may be specifically increased. HU affects the expression of genes for matrix deposition and maturation, BMP1 and PCOLCEs, in the growth plate and in the bone cells, suggesting a lesser formation of cross linked collagen fibers and impaired bone matrix maturation.

Changes in the pattern of expression observed in cells of the distal bone marrow for genes involved in bone homeostasis, Stro1, CXCR4 and CXCL12, suggest that complex changes in bone homeostasis may occur in HU mice, apparently not involving CD146 expression.

Return to pre-treatment levels of gene expression for most genes studied occurs after the mice in HU for 14 days were set on the ground again for 14 days, with the exception of AP in all cells and CXCR4/CXCL12 in bone marrow at the distal epiphysis.

## Methods

### Hindlimb unloading

11 week old C57BL/6J male mice (Charles River, Italy) were suspended for the tail in a special cage [31,32], a condition referred as HU, for 3, 7 or 14 days. Food and water were ad libitum. Control of the amount of food uptake showed no differences at any time among the groups of animals in different conditions. In each experiment 3 to 6 mice were HU for each time point and the same number were kept on the ground as control. Re-loading of the hind limb occurred by returning mice to standard conditions for 14 days after HU. Animal treatment was in accordance with the Italian Guidelines for the use of laboratory animals, which conforms with the European Community Directive published in 1986 (86/609/EEC).

### Microcomputed Tomographic (μCT) Analysis

Whole femora (one per mouse) were examined by a μCT system (Desktop μCT 40, Scanco Medical AG, Bassersdorf, Switzerland) at the Bone Laboratory of The Hebrew University of Jerusalem, courtesy of Prof. I. Bab and Dr. A. Bajayo, as reported previously [33]. Scans were performed at a 20 μm resolution in all three spatial dimensions and all morphometric parameters were determined using a direct 3-D approach. Three preselected regions: 1) whole bone; 2) secondary spongiosa in the distal metaphysis extending proximally 3 mm from the proximal tip of the primary spongiosa; 3) a diaphyseal segment extending 1 mm distally from the midpoint between the femoral ends. For the whole bone we determined the Apparent Volume Density (AVD) [34]. Parameters determined in the metaphyseal trabecular bone included bone volume density (BV/TV), trabecular thickness (Tb.Th), trabecular number (Tb.N) and trabecular connectivity (Conn.D). Cortical thickness (Cort.Th), diaphyseal diameter (Dia.Dia) and the medullary cavity diameter (Med.Dia) were determined in the mid-diaphyseal region.

### Preparation for immunohistochemistry and in situ hybridization

Tibiae were dissected free of soft tissues and fixed in PAF 4% in PBS 1× a 4°C for 48 hours, decalcified with Osteodec (Bioptica, Italy) for 16 hours, dehydrated by successive alcohol passages and xylol washing and embedded in paraffin at 58°C. Sections at the microtome were 4 μm thick and the sections in the central portion of the bone were stained with H.E. or used for immunohystochemistry and in situ hybridization.

### Immunohistochemistry

De-paraffinized sections were processed by citrate unmasking of the antigens at 95°C, peroxidase quenching, rinsed in PBS and incubated with the primary antibody for 60' at room temperature, followed by rinses and incubation for 60' with the secondary antibody (HRP polymer conjugate broad spectrum DAB kit from Zymed Laboratories inc., cat. 87-9663). Counterstaining was with Hematoxylin. Controls were run omitting the primary antibody. Primary antibodies (all utilized at 1/50 dilution) were: CXCL12 polyclonal in rabbit (sc-28876, Santa Cruz), RECK polyclonal in rabbit (sc-28918, Santa Cruz), BSP polyclonal in rabbit (LF87, kindly donated by Prof. L.W. Fisher, Bethesda, USA), BMP-1 polyclonal in rabbit (sc-33200, Santa Cruz, USA, recognizing all splicing variants and Tll1, Tll2 protein), OC polyclonal in rabbit (sc-30045, Santa Cruz, USA), CD146 polyclonal in rabbit (sc-28667, Santa Cruz, USA) and Stro-1 mouse ascites (kindly donated by Prof. Ranieri Cancedda, CBA, Genoa, Italy). We show exemplary micrographs from at least two experiments of HU and 2 or 3 mice for each condition from each experiment, which gave consistent results.

### In situ hybridization

Probes utilized were: AP (NM_007431) 600 bp cDNA cloned in PBSSK plasmid, kindly donated by Prof. Tuan; OC (NM_007541) 350 bp cDNA cloned in PBSSK plasmid, kindly donated by Prof J.E. Aubin (recognizing all three genes including in the osteocalcin cluster [35]); MT1-MMP (NM_008608) 1021 bp cDNA cloned in PBSSK, kindly donated by Prof. Lewalle; BMP1 (NM_009755) 827 bp cDNA cloned in PSP65 plasmid, kindly donated by Prof. Wozney (recognizing BMP1 and all variants differently spliced); MMP-9 (NM_013599) 676 bp cDNA cloned in PUC19 plasmid, kindly donated by Prof. W.G. Stetler-Stevenson: VEGF-A, 700 bp cDNA cloned by RT-PCR with the PCR TA cloning kit (Invitrogen, USA) from human total RNA in PCR 2.1 vector (primers for human VEGF-A: Lp CCTTGCTGCTCTACCTCCAC Rp TCTGTCGATGGTGATGGTGT, recognizing all VEGF-A mRNA splicing variants); CXCR4 (NM_009911) 692 bp cDNA cloned by RT-PCR with PCR-Script Amp cloning kit (Stratagene, USA) from human total RNA in pPCR-script amp SK(+) vector (primers for human CXCR4: Lp ATGCAAGGCAGTCCATGTCAT Rp TCTGTCGATGGTGATGGTGT [36], recognizing the two mRNA splicing variants) and PCOLCE (NM_008788) 501 bp cDNA cloned by RT-PCR from rat total RNA in PBSSK vector (primers for rat PCOLCE: Lp ATGCTCTGGAGGTCTTTGCT Rp TGAGATCTGATACAAACTGG, recognizing the genes for PCOLCE1 and PCOLCE2). Linearized plasmids were used with dig-labeling nucleic acids kit (Boeringher-Mannheim, Germany) to obtain sense or antisense digoxigenin-labeled riboprobes from the appropriate promoter. Hybridization reactions were performed on serial sections of 4 μm thick, de-paraffinized, dehydrated and post-fixed with 4% PAF pH 9.5 in PBS 1× [37]. After digestion with HCl 0.2 M and Proteinase K 10 μg/ml at 37°C, hybridization was in 3× SSC, 1 mg/ml tRNA, 10 mM DTT, Denhardt's solution 1×, 50% formamide, 1 mg/ml denatured salmon sperm, 10% dextran sulfate and 1 ng/μl of the denaturated probe, maintained overnight at high stringency. Temperatures of annealing and stringency of washes were optimized for each probe [38]. The slides were washed in 2× SSC, digested with RNAse A 100 μg/ml at 37°C, incubated overnight at 4°C with antidigoxigenin Fab fragments conjugated to alkaline phosphatase (Boeringher-Mannheim, Germany) in buffer with 1.5 M NaCl, 0.1 M TRIS-HCl, 2 mM MgCl2, 0.3% Triton X-100, 10% fetal calf serum, pH 7.5. Color development was in buffer 0.1 M NaCl, 0.1 M TRIZMA base, 5 mM MgCl_2_, 10% polyvinyl alcohol 89-98 kDa (Sigma, USA), 1 mM levamisole (Sigma, USA), 0.16 mg/ml BCIP, 0.33 mg/ml NBT (Roche, Germany), pH 9.5, for 3-6 hours at 30°C [39]. Slides were counterstained with nuclear fast red 0.005% and mounted in glycerol gelatin (Sigma, USA). We show exemplary micrographs from at least two experiments of HU, and 3 mice for each condition and from each experiment, which gave consistent results.

### Image acquisition

The images were captured with a Leica DMBR microscope mounted with a Leica camera DFC320 and acquired with Leica Firecam version 1.9.1.

### Statistics

For mice weight a one-way ANOVA test was performed to define significance.

## Results

**Figure 1:**
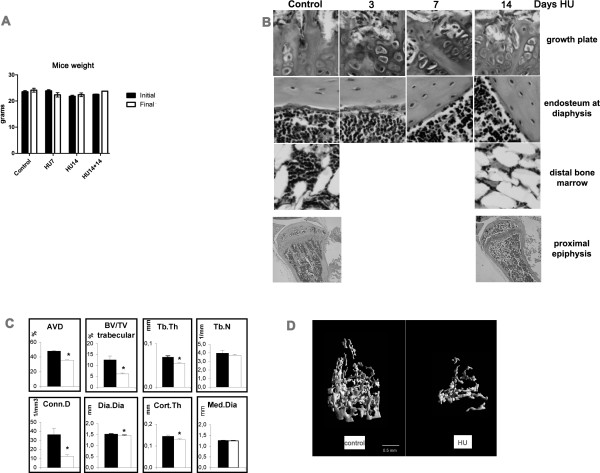
**Growth unaffected in HU mice, and changes in bone histomorphometry**. A- Mice weight (mean +/- SEM). The values are obtained from the measure of the weight of 6 mice per point for each condition, below. Means +/- SEM. One-way ANOVA shows no significant difference for p < 0.05. B- Photomicrographs of Hematoxylin-Eosin. Stained sections from tibiae of mice at different days in HU were taken at the growth plate, cortical bone at mid-diaphysis, bone marrow in the distal region of the tibia. Enlargement is 200×. On the bottom the whole of the proximal epiphysis, 40×. C- Bone morphometric parameters by μCT, in control and HU14 mice. Apparent volume density (AVD), trabecular bone volume density (BV/TV trabecular), trabecular thickness (Tb.Th), trabecular number (Tb.N), connectivity density (Conn.D), diaphyseal diameter (Dia.Dia), cortical thickness (Cort.Th), medullary cavity diameter (Med.Dia). Data are mean ± SEM obtained in 6 mice per condition. *, p < 0.05. Black columns control, white columns HU14. D- 3D μCT femoral images from control and HU for 14 days.

We examined 3 mice for each time point of HU in each of 2 or 3 separate experiments compared with equal numbers of control mice. No significant differences in body weight were found in HU mice compared with age matched control mice during all the experimental periods, nor with mice kept in HU for 14 days plus 14 days after reloading of hind limbs (Figure 1A). This shows lack of significant growth during the experimental time and suggests lack of stress due to the HU condition.

Figure 1B shows the morphology of growth plate, diaphyseal endosteum and bone marrow in the distal epiphysis of controls and in mice kept for 3, 7 or 14 days in HU. 3 days after HU, the cartilage at growth plate already shows a disrupted columnar arrangement, with loss of the connection to the morphologically unchanged trabecular organization underneath. Osteogenic cells, here shown lining the endosteum at diaphysis, but equally on trabeculae (not shown), became disorganized and fewer after 7 days of HU. At this time no obvious thinning of the diaphyseal bone was detected. 14 days after HU, the trabeculae appear severely disarranged and have reduced thickness. This observation is more evident in the micrograph at low enlargement including the whole proximal epiphysis. Bone marrow in the distal epiphysis shows decrease in cellularity after 14 days in HU, which might be due to increase in size/number of adipocytes.

Quantitative μCT analysis shows significant HU-induced decreases in total bone volume (AVD, 26%) which is the percent mineralized tissue volume over the total bone volume defined by the external bone envelope. Also decreased are trabecular bone volume (51%), trabecular thickness (20%), and connectivity density (66%) at 14 days in HU. The diaphyseal diameter shows a 57 μm decrease (28.5 μm in diaphyseal radius). In the absence of a parallel significant reduction in the medullar cavity diameter, this decrease results in 16 μm thinning of the cortex (Figure 1C). Substantial cortical and trabecular bone loss, with disorganization of trabecular architecture, is evident after 14-days HU (Figure 1D).

We examined gene expression by in situ hybridization and protein expression by immunohistochemistry. We could not detect changes in gene expression in mice after 3 days of HU. We here report, summarized in Table 1, the changes observed in gene expression in mice after 7 and 14 days of HU in comparison with matched age controls. Expression levels in control mice remained constant at each time within the 28 day experimental period, and so for each of the figures below, we only show the data from controls at 14 days. In each of the figures images show in sequence from left to right in the panel columnar pre-hypertrophic chondrocytes, osteoblasts on endosteum and on trabeculae, in bone marrow cells in the central diaphysis and in the distal epiphysis.

**Table 1:** Gene and protein expression in HU mice and recovery versus control.

mRNA Vs. control	HU7	HU14	HU14+R14	expressed in
AP	↓	↓	↓	growth plate chondrocytes, osteogenic, bone marrow cells

OC	↓	=	=	growth plate chondrocytes, osteogenic, bone marrow cells

MT1-MMP	↑	↑	=	growth plate chondrocytes, osteogenic, bone marrow cells

MMP9	=	↓	=	growth plate chondrocytes, osteogenic, bone marrow cells, macrophages

BMP1	= (ns)	↓	=	growth plate chondrocytes, osteogenic, bone marrow cells

PCOLCEs	=	↓	= (↓distal bone marrow)	growth plate chondrocytes, osteogenic, bone marrow cells

CXCR4	= (ns)	↑	=	osteogenic, bone marrow cells

				

**Protein vs. control**	**HU7**	**HU14**	**HU14+R14**	**expressed in**

OC	ND	↓	=	growth plate chondrocytes, osteogenic, bone marrow cells

BSP	↓	= (↓osteoblasts)	ND	osteogenic, bone marrow cells

RECK	↓	=	ND	osteogenic cells

BMP1	= (ns)	↓	=	growth plate chondrocytes, osteogenic, bone marrow cells

Stro1	↑	=	ND	osteogenic, bone marrow cells

CXCL12	ND	↓	= (only osteoblasts)	bone marrow cells

Arrows pointing upwards indicate increase, pointing down indicate decrease of expression related to the corresponding control; = stays for equal to control. ND, not done; ns, not shown in the corresponding figure.

**Figure 2:**
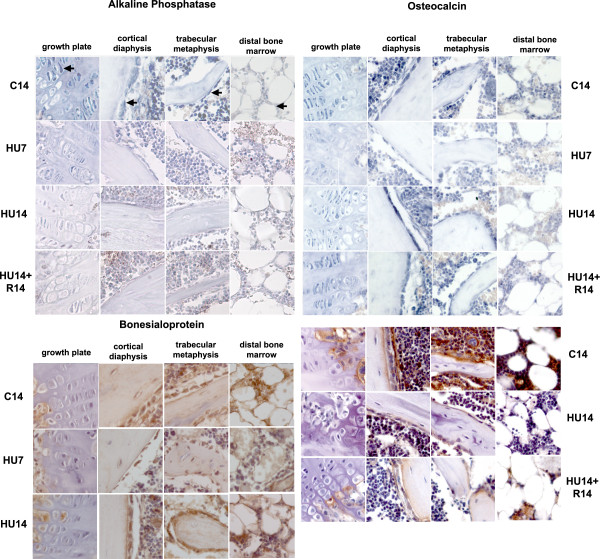
**Changes in the pattern of expression of genes involved in the osteogenic progression (AP, OC and BSP) induced by HU, and their reversibility**. Gene expression analyzed by in situ hybridization for AP, OC and by immunohistochemistry (for OC and BSP). Control mice at 14 days (C14) and mice in HU for 7 (HU7) or 14 days (HU14) and for 14 days in HU, followed by 14 days of re-loading (HU14+R14). From left to right in each row: growth plate, cortical bone at mid-diaphysis, trabecular bone in the proximal region, bone marrow in the distal region of the tibia. Arrows in the first lane point to different cell types in these compartments, respectively pre-hypertrophic columnar chondrocytes, osteogenic cells of the endosteum, osteogenic cells lining trabeculae and bone marrow cells. Probes are described in M&M. Enlargement is 200×.

Among the genes characterizing the osteogenic progression, the mRNA for AP (Figure 2, top-left) is expressed in control mice in all these cells, with lesser intensity in those in the distal epiphysis. Its amount decreases severely after 7 days of HU (HU7) and remains low at 14 days of HU (HU14) in pre-hypertrophic chondrocytes, osteoblasts on endosteum, and a smaller decrease is detected in bone marrow cells at the distal epiphysis. The level of AP mRNA does not return to control level in HU14 mice recovering on the ground for 14 days (HU14+R14).

The three genes included in the OC cluster detected by our RNA probe are expressed in the same cell types expressing AP (Figure 2, top-right). The level of OC mRNAs is transiently decreased in all cell types after 7 days of HU, and returns to control level during the second week of HU. The transient decrease of mRNA in HU mice is reflected on the protein level (immunohistochemistry in Figure 2, bottom-right) which is lower than controls after 7 days (not shown), remains low up to 14 days of HU, and partly increases after recovery.

The expression of BSP protein (Figure 2, bottom-left) is restricted to osteogenic and bone marrow cells. Compared to controls, the protein level transiently decreases after 7 days of HU and returns to control levels after 14 days of HU.

**Figure 3:**
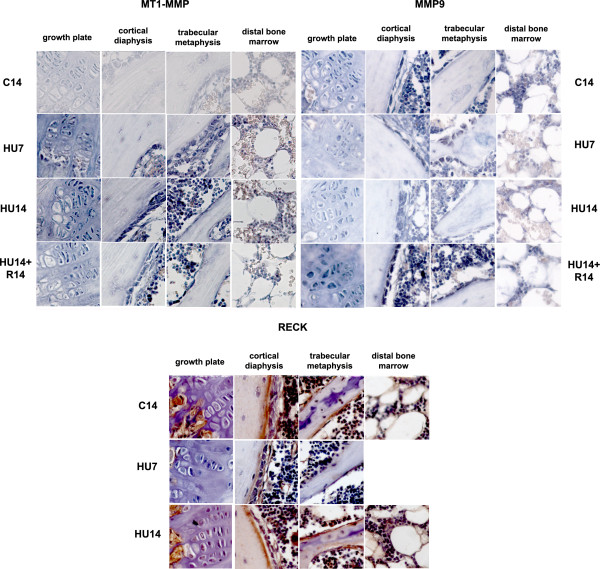
**Changes in the pattern of expression of genes involved in ECM proteolysis (MMP-9, MT1-MMP, RECK) induced by HU, and their reversibility**. Gene expression analyzed by in situ hybridization for MT1-MMP and MMP9 and immunohistochemistry for RECK. Description as in legend of Figure 2. N.B. one micrograph is missing from the panel due unsuitable photographic quality of the sections. The experimental data is available and was readable by direct microscopic inspection.

MT1-MMP expression occurs in the same cell types as those expressing AP, and its mRNA increases in HU7 and HU14 mice (Figure 3, top-left). The level of gene expression decreases after recovery. All the cell types that express MT1-MMP show a similar pattern and timing of change in gene expression during HU and after recovery.

MMP-9 mRNA (Figure 3, top-right) is expressed in chondrogenic, osteogenic, bone marrow mesenchymal cells and macrophages. The expression of the transcript is progressively decreased during the two weeks of HU, compared to control, and returns to a level similar to control after recovery.

The expression of the protein for the endogenous inhibitor of MMPs, RECK (Figure 3, bottom) is observed in osteoblasts and in mesenchymal cells under the growth plate. The amount of RECK transiently decreases after 7 days of HU in all cell types, returns to control levels after 14 days of HU, and no further changes occur after recovery for 14 days (not shown).

**Figure 4:**
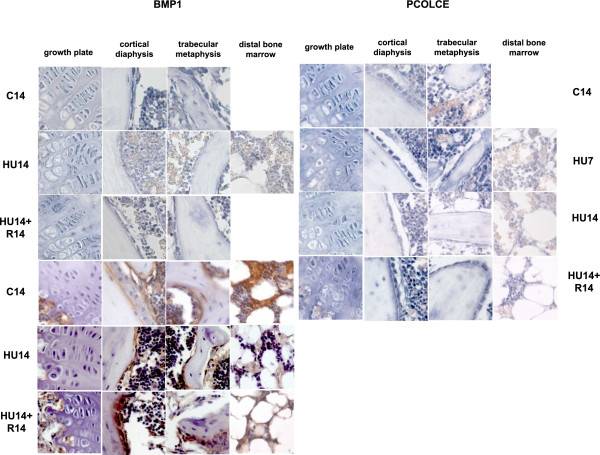
**Changes in the pattern of expression of genes involved in ECM deposition (BMP1, PCOLCE 1, 2) induced by HU, and their reversibility**. Gene expression analyzed by in situ hybridization for BMP1 and PCOLCE 1, 2. Description as in legend of Figure 2. For micrographs missing from the panel see legend Figure 3.

The expression of BMP1 and PCOLCE1, 2 occurs in the same cell types expressing AP. 14 days after HU is detected down modulation in the levels of expression for mRNAs (Figure 4, top-left) and proteins (Figure 4, bottom-left), and the mRNA level is restored after recovery for 14 days.

The modulation of expression of mRNAs for the PCOLCE 1,2 (Figure 4, top-right), is similar to that of BMP1, with a decrease detected after 14 days of HU, and returning to control level after recovery for 14 days.

**Figure 5:**
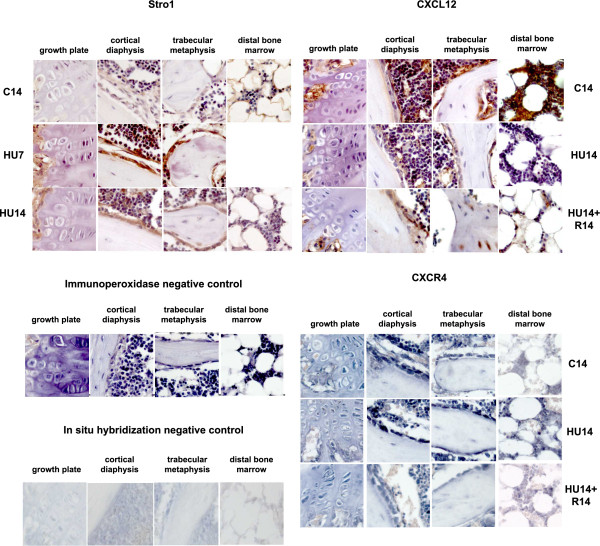
**Changes in the pattern of expression of genes involved in bone homeostasis (Stro1, CXCL12 and CXCR4) induced by HU, and their reversibility**. Gene expression analyzed by immunohistochemistry for Stro1, CXCL12 and by in situ hybridization for CXCR4. Description as in legend of Figure 2. For micrographs missing from the panel see legend Figure 3. Control for immunohistochemistry, without primary antibody, and for in situ hybridization, here with sense probe for MT1-MMP, are shown respectively in the second and fourth panel from the top.

Stromal protein Stro1 (Figure 5, top-left) is expressed in osteogenic and bone marrow cells and is transiently up modulated after 7 days of HU.

The CXCL12 protein is detected in controls with highest expression in the bone marrow cells (Figure 5, top-right) and is down modulated in HU14 mice; after recovery for 14 days the protein is detected again in bone cells and also in the osteocytes within the trabeculae, but its expression is not restored in the cells of the distal bone marrow.

The expression of the mRNA for the CXCL12 receptor, CXCR4, is detected in bone and bone marrow cells (Figure 5, third panel from the top), is highly up modulated in the bone marrow at distal epiphysis after 14 days of HU and only slightly decreases after recovery.

Immunostaining for the stromal protein CD146 and in situ hybridization for VEGF (with a probe detecting the 3 forms of VEGF-A) were also done, and these genes did not show significant differences between control and HU mice (not shown).

## Discussion

We report the evidences of modulation of the expression of genes and proteins in mesenchymal cells of mice tibia during HU. We also report the restoration of control gene and protein expression after re-loading of the limbs for 14 days.

Although in HU rodents the osteoblasts and mesenchymal osteogenic precursors were identified as the first cellular component responding to the lack of weight bearing with increased apoptosis [3], the effects of HU on gene expression at the cellular level in mesenchymal cells was not previously studied [5-9] and no information was available about how HU affects the expression of genes involved in the deposition, organization and remodeling of ECM.

We investigated either gene and/or protein expression, according to the availability of probes, as summarized in Table 1.

We show that HU can modulate the expression not only of genes involved in osteogenesis, but also of genes involved in ECM proteolysis and deposition, and of genes involved in the homeostasis of bone.

The modulation by HU of many genes and proteins occurs at the same time and with the same sign (increase or decrease of expression) in the cells residing in different tissue microenvironments of the long bone, with the exceptions described below for bone marrow in the distal epiphysis.

In control mice co-expression of AP, MT1-MMP, OC, MMP-9, PCOLCEs and BMP1 occurs in chondrogenic, osteogenic and bone marrow mesenchymal cells, while BSP, Stro1, VEGF-A and CD146 (these last two not shown) and CXCR4 expression are restricted to osteogenic and bone marrow cells. RECK expression is localized to osteoblasts at the endosteum and under the growth plate, and CXCL12 expression to bone marrow cells.

No changes were detected after 3 days of HU but changes in gene and protein expression were evident by 7 days as decreases in the levels of AP, OC, BSP, RECK, and increases in the levels of MT1-MMP and Stro1. At this time disorganization of the trabecular arrangement at the growth plate and changes in the morphology of the osteoblasts lining the endosteum were already evident, but no obvious thinning of the diaphyseal bone was detected.

After 14 days of HU, bone erosion was measured and decreases in the level of expression of MMP 9, BMP1 and PCOLCEs, CXCR4, CXCL12 were detected. The changes in gene expression observed after 7 days of HU persisted and/or were enhanced for some genes, (as the decrease in AP and the increase in MT1-MMP), while for other genes and proteins the level of expression reverted to that of the control (as for OC, BSP, RECK, Stro1).

Recovery on the ground for 14 days, after 14 days of HU, determined return to control level and reversal of the changes induced by HU, for expression of all the genes and proteins in chondrocytes at the growth plate and for osteoblasts and bone marrow cells in proximal and mid tibiae, with the exception of AP. This observation is in agreement with the slow recovery of AP expression after HU in rodents, in man and other mammals after space flight.

Whereas AP expression decreased and was not reversible by re-loading, MT1-MMP expression increased during HU and returned to control by 14 days after limb re-loading. This evidence suggests that the positive pleiotrophism that couples MT1-MMP and AP expression during osteogenesis [22] was disrupted during HU.

In general, and with the exceptions below, synchrony and similarity in direction of the changes induced by HU and occurring upon recovery were observed in all the mesenchymal cells of different lineages and in the context of different local microenvironments. This suggests that a common mechanism for response to mechanical forces is activated in all cell types, and mediates the sensing of the changes induced by unloading, causing responses at cellular level. This mechanism would be independent from the different composition and characteristic of the local ECM, such as in cartilage, bone or bone marrow.

We suggest that MT1-MMP, expressed in all the cell types residing in these different ECMs, may play a key role in sensing the mechanical changes induced by HU. MT1-MMP expression is know to be modulated by mechanical forces [39], and biochemical microenvironment [40], and has the potential to "sense" the interaction with ECM [18], which in turn can affect the expression of other genes.

The increase in pericellular proteolysis, in pro-MMP-2 activation and in MT1-MMP self-regulation, ensuing from MT1-MMP up modulation by HU, can play a key role in the remodeling of ECM during HU, in association with the decrease of the activity of MMP-9. This may determine the establishment, and possibly the maintenance, of a novel pericellular microenvironment. At the same time, transient down modulation of BSP and RECK in the osteogenic cells can specifically affect the organization of mature bone ECM. These events might be associated to the early apoptotic response of osteogenic cells to HU. The early transient up modulation of Stro1 might reflect the response to HU of cells in the mesenchymal compartment of bone marrow.

The decrease in procollagenase and PCOLCEs activities, occurring only after 7 days of HU, may then diminish collagen fibrillogenesis and deposition of the novel ECMs of the several kinds present in the long bone, contributing to the loss of bone density.

The effects of HU on CXCR4 and CXCL12 in the tibia are specifically localized to the compartment of distal bone marrow. In general, this compartment has a distinct response by comparison to the diaphyseal bone marrow, e.g. it is preferentially enriched in adipocytes in HU animals and in other models of induced osteoporosis [41,42].

A specific pattern of HU-induced modulation was found for the CXCL12 protein, highly expressed in the bone marrow cells, which is down modulated after 14 days of HU. CXCL12 level was restored after recovery in osteoblasts and in the osteocytes within the trabeculae, but not in the bone marrow. CXCR4, the CXCL12 receptor, mRNA was up modulated after 14 days in HU, particularly in the cells of the bone marrow in the distal tibia, where it continued to be expressed at a relatively high level also after recovery. The effects of HU on cells in the bone marrow in the distal epiphysis might unbalance the CXCR4/CXCL12 signaling at least regionally. In view of the role that this signaling pathway plays in bridging hematopoiesis, osteogenesis and osteoclastogenesis, these effects might be relevant in the homeostasis of the whole bone. Since CXCL12 expression did not return to control level in the distal bone marrow after recovery, the unbalance might persist and, the restoration of bone balance might lag.

In this report we have not mentioned gene expression in multinucleated cells in the bone marrow and in osteoclasts; these are part of an ongoing study. We had problems focusing on osteocytes at the microscope, possibly due to the relatively thick sections obtained from paraffin embedded whole bones, which limited our report about this cell type.

## Conclusion

To summarize, the net effects at the cellular level of HU treatment in mice were to depress the expression of genes for osteogenic progression, to uncouple the expression of AP and MT1-MMP, to decrease generalized ECM degradation via down modulation of MMP-9, and to increase the pericellular proteolysis due to increased expression of MT1-MMP, in the presence of transiently decrease of the MMPs-inhibitory protein RECK in the osteogenic cells. Anabolic genes that govern collagen synthesis and deposition in fibrils (procollagenases and their enhancer proteins) were for the first time identified as targets of HU-dependent inhibition in cartilage, bone marrow and bone cells. This inhibition possibly aggravates the loss of matrix mass, already known to occur during HU, due to the reduction in collagen synthesis. The down-modulation of these functions may also contribute, together with the transient decrease of BSP protein in osteogenic cells, to the specific impairment of the maturation of osteogenic matrix.

The simultaneous timing and direction of the response to HU for genes involved in the osteogenic progression and genes involved in proteolysis and deposition of the ECM occurring in cells of different bone compartments and lineages, suggests that it might exist a common process of sensing the reduced gravity. We suggest that MT1-MMP increase is a key element in this response, able to mediate and influence further changes during HU. The response to HU for specific genes involved in homeostasis and localized to cells of the distal bone marrow points the possibility that HU might affect the homeostatic balance of the whole bone; this point requires further studies in vitro and in vivo.

No changes were detected in CD146 protein and for VEGF-A.

Limb re-loading caused recovery of the previous pattern of expression with the exceptions of AP in the whole bone, and of CXCL12/CXCR4 in distal bone marrow. This suggests that, at least in the experimental time analyzed, after re-loading of the limbs "*all will not return as well as before*". Further investigation is particularly important to define if and when the expression of these genes is recovered and how it is relevant for the re-acquisition of bone mass after re-loading.

## Competing interests

The authors declare that they have no competing interests.

## Authors' contributions

DV was the main research fellow, who performed the HU experiments, collected the samples of bones, prepared the molecular probes and performed and the in situ hybridization. He also contributed in writing the manuscript. AS did the histological preparation of the bones, and their sectioning. She took care of the immunohistochemistry experiments. DP contributed to the blind reading of the in situ hybridization and immunohistochemistry slides, she also participated in selecting and editing the figures and to the final editing of the manuscript. PM designed and supervised the experiments, coordinated the project and wrote the manuscript. All authors read and approved the final manuscript.

## Pre-publication history

The pre-publication history for this paper can be accessed here:

http://www.biomedcentral.com/1471-2474/11/147/prepub
